# Association between dietary niacin intake and risk of Parkinson’s disease in US adults: cross-sectional analysis of survey data from NHANES 2005–2018

**DOI:** 10.3389/fnut.2024.1387802

**Published:** 2024-07-18

**Authors:** Ling Zhang, Shaojie Yang, Xiaoyan Liu, Chunxia Wang, Ge Tan, Xueping Wang, Ling Liu

**Affiliations:** ^1^Department of Neurology, West China Hospital of Sichuan University, Chengdu, China; ^2^Department of Neurology, Chengdu Eighth People’s Hospital (Geriatric Hospital of Chengdu Medical College), Chengdu, China; ^3^Department of Neurology, The First People’s Hospital of Longquanyi District, Chengdu, China; ^4^Department of Neurology, 363 Hospital, Chengdu, China; ^5^Mental Health Center, West China Hospital of Sichuan University, Chengdu, China; ^6^Department of Neurology, The First Hospital of Lanzhou University, Lanzhou, Gansu, China

**Keywords:** Parkinson’s disease, niacin, vitamin B3, National Health and Nutrition, NHANES

## Abstract

Parkinson’s disease (PD) is one of the most common neurodegenerative diseases and involves various pathogenic mechanisms, including oxidative stress and neuroinflammation. Niacin, an important cofactor in mitochondrial energy metabolism, may play a key role in the pathogenesis of PD. An in-depth exploration of the relationship between niacin and mitochondrial energy metabolism may provide new targets for the treatment of PD. The present study was designed to examine the association between dietary niacin intake and the risk of PD in US adults. Data from adults aged 40 years and older collected during cycles of the United States (US) National Health and Nutrition Examination Survey (NHANES) from 2005 to 2018 were used. A multiple logistic regression model was used to analyze the relationship between dietary niacin intake and the risk of PD. Further linear tests using restricted cubic splines (RCS) were performed to explore the shape of the dose–response relationship. Subgroup stratification and interaction analyses were conducted according to years of education, marital status, smoking, and hypertension to evaluate the stability of the association between different subgroups. A total of 20,211 participants were included in this study, of which 192 were diagnosed with PD. In the fully adjusted multiple logistic regression model, dietary niacin intake was negatively associated with the risk of PD (OR: 0.77, 95%CI: 0.6–0.99; *p* = 0.042). In the RCS linear test, the occurrence of PD was negatively correlated with dietary niacin intake (nonlinearity: *p* = 0.232). In stratified analyses, dietary niacin intake was more strongly associated with PD and acted as an important protective factor in patients with fewer years of education (OR: 0.35, 95%CI: 0.13–0.93), married or cohabitating (OR: 0.71, 95%CI: 0.5–0.99), taking dietary supplements (OR: 0.6, 95%CI: 0.37 0.97), non-smokers (OR: 0.57, 95%CI: 0.39–0.85), those with hypertension (OR: 0.63, 95%CI: 0.63–0.95), coronary artery disease (OR: 0.77, 95%CI: 0.6–1), and stroke (OR: 0.75, 95%CI: 0.88–0.98), but the interaction was not statistically significant in all subgroups. Dietary niacin intake was inversely associated with PD risk in US adults, with a 23% reduction in risk for each 10 mg increase in niacin intake.

## 1 Introduction

Parkinson’s disease (PD) is one of the most common neurodegenerative disorders, and is primarily caused by the loss of dopamine-producing neurons in the substantia nigra (1, 2). It affects over 6 million people worldwide and is a leading cause of neurofunctional impairments (3, 4). The pathogenic mechanisms of PD involve multiple aspects, with mitochondrial dysfunction, oxidative stress, and neuroinflammation as the crucial core mechanisms (5–8). Currently, no cure for PD (9) exists, and understanding its pathogenic mechanisms and identifying new drug targets for treatment and prevention is of paramount importance.

Niacin, also known as vitamin B3, is a precursor to nicotinamide adenine dinucleotide (NAD) and nicotinamide adenine dinucleotide phosphate (NADP) (10), possesses anti-inflammatory properties, enhances mitochondrial function by supplying NAD (7, 11), and serves as an essential cofactor in mitochondrial energy metabolism (12). Lack of niacin in the diet may disrupt mitochondrial respiration and reduce oxidative phosphorylation (13). Some studies have suggested that niacin is beneficial in the treatment of PD by alleviating inflammation through an NIARC1-related mechanism and increasing dopamine synthesis in the striatum by supplying NADPH to the mitochondria (14). Research has explored niacin treatment for patients with PD, suggesting a potential role in symptom alleviation and disease progression delay (15–17). However; to date, no studies have been conducted in the general population to investigate the association between niacin and the risk of PD. Therefore, this study aims to evaluate the relationship between dietary niacin intake and the risk of PD in American adults using data from the National Health and Nutrition Examination Survey (NHANES). The specific objective is to determine whether higher dietary niacin intake is negatively associated with the risk of PD.

In a large cross-sectional study of American adults aged 40 and above conducted from 2005 to 2018, we hypothesize that higher dietary niacin intake may be associated with a lower risk of PD. We hope this study will provide stronger evidence for the role of niacin in PD prevention.

## 2 Materials and methods

### 2.1 Data source

This cross-sectional observational study utilized data from the NHANES website. The NHANES is a multistage, large, stratified, and nationally representative study of the US population that provides detailed information about study design, interviews, and demographics, etc. (18–20). The present study was reviewed and approved by the National Institute of Public Health Research Ethics Committee. Written informed consent was obtained from the participants’ legal guardians or close family members (21).^1^ To address potential sources of bias, the NHANES database implemented standardized procedures during data collection, and data collectors received comprehensive training to ensure consistency and accuracy, thereby reducing information bias. Individuals aged 40 and above who completed the interviews participated in our study. We excluded pregnant individuals and those with missing dietary niacin intake and covariate data.

### 2.2 Diagnosis of PD

Consistent with previous literature (22–24), participants were considered to have PD when using “anti-Parkinson’s agents” based on answers to questions about prescribed medications. Owing to the limitations of drug and code inclusion in the NHANES, patients must be treated with Parkinson’s drugs to be classified as having PD, whereas others are classified as non-PD.

### 2.3 Dietary niacin intake

Dietary intake data were collected by trained dietary interviewers using the NHANES Computer-Assisted Dietary Interview (CADI) system. Each Mobile Examination Center (MEC) dietary interview room follows a set of standardized measurement guidelines, which are agreed upon by experts during regular workshops and specifically designed for the current NHANES setting. These guidelines assist respondents in accurately reporting the quantity and portion size of consumed foods. The NHANES Dietary Interview Procedures Manual provides a comprehensive overview of the dietary interview methodology (25).

The database employs the multiple-pass recall method to gather food information, offering two dietary niacin intake recalls, both reflecting intake within a 24 h period. The first recall is conducted at the NHANES MEC, and the second recall is completed via telephone interview on days 3–10 following the first recall (24). To ensure data accuracy, the average of the two dietary niacin intake recalls was used as the final intake value. Niacin in this study refers to dietary niacin and excludes niacin supplements.

### 2.4 Covariates assessment

The covariates in this study were based on previous literature (20, 26), and a variety of possible covariates were evaluated including age, sex, race, marital status, family income, education level, body mass index (BMI), smoking status, dietary supplements, calorie consumption, carbohydrate consumption, protein consumption, and fat consumption. Chronic comorbidities included diabetes, hypertension, coronary heart disease, and stroke. Marital status was defined as living alone or with a partner. Educational levels were divided into three groups based on the 9-year and 12-year boundaries. Races were classified as Mexican American, non-Hispanic black, non-Hispanic white, and other. Sixty-five years old is commonly regarded as the dividing line between middle age and old age. In this study, participants were divided into middle-aged and elderly groups based on this cutoff point. According to a US government report, family income was classified as low, middle, and high based on a poverty income ratio (PIR) of 1.3 and 3.5 (25). Smoking status was determined based on questionnaire responses, following definitions from previous literature (27, 28). Individuals who have smoked more than 100 cigarettes in their lifetime were categorized accordingly: those who smoked fewer than 100 cigarettes were classified as non-smokers; current smokers were individuals who currently smoke and have smoked more than 100 cigarettes in their lifetime; former smokers were individuals who have smoked more than 100 cigarettes in the past but have since quit. Chronic comorbidities were obtained through questionnaires, which inquired whether participants had been diagnosed with these diseases by a doctor. We selected four chronic conditions with a high prevalence rate (diabetes, stroke, hypertension, and coronary heart disease) as the chronic comorbidities for this study. BMI was calculated by dividing weight by the square of height, and participants were categorized into normal weight and overweight groups based on the standard proposed by the World Health Organization, with 25 kg/m^2^ as the cutoff point. The participants’ total dietary calories, fat, protein, and carbohydrate values were obtained through dietary recall. Information on dietary supplements was also obtained through dietary recall from questionnaires regarding whether the participants had taken dietary supplements in the past month.

### 2.5 Statistical analyses

In this study, the Kolmogorov–Smirnov test was used to determine whether continuous variables were normally distributed. The mean value (standard deviation) was used to represent normally distributed variables and the median (interquartile distance) was used to represent skewed variables. Categorical variables were expressed as percentages. One-way analysis of variance (ANOVA) was used for normal distributions, the Kruskal-Wallis test for skewed distributions, and the Chi-square test was used for categorical variables. Odds ratios (OR) and 95% confidence intervals (95%CI) between dietary niacin intake and PD were calculated using logistic regression models. Due to dimensional problems, when this analysis was performed using niacin as a continuous variable, we divided its value by 10 in units of 10 mg per unit. Model 1 was adjusted for uncontrollable sociodemographic characteristics including age, sex, and race. Model 2 was adjusted for all sociodemographic characteristics and all covariates other than chronic comorbidities including age, sex, race, education level, marital status, family income, BMI, smoking status, calorie consumption, protein consumption, carbohydrate consumption, fat consumption, and dietary supplements. Model 3 was adjusted comprehensively to include chronic comorbidities (hypertension, coronary heart disease, stroke, and diabetes) based on Model 2.

We used a restricted cubic spline (RCS) test to determine the shape of the dose–response relationship between dietary niacin intake and the incidence of PD. Four nodes of dietary niacin level distribution (at the 5th, 35th, 65th, and 95th percentiles) were used to build a smooth curve-fitting plot according to all covariables included in Model 3. Subgroup analyses of sex, age, race, marital status, education, smoking status, family income, BMI, dietary supplements, hypertension, coronary heart disease, stroke, and diabetes were performed using logistic regression models. Interactions between subgroups were tested using the likelihood ratio test (P for interaction). To assess the robustness of the results, we excluded participants with extreme energy consumption, specifically those with a daily energy consumption of <500 or >5,000 kcal, for a sensitivity analyses.

As the sample size of this study was completely dependent on the NHANES database, no statistical performance estimation was performed in advance. The study excluded all missing data, so there is no data missing. In this study, R open-source software version 4.0.4 and Free Statistics software (29) version 1.9 were used for statistical analyses. We conducted a descriptive study of all participants, and a *p*-value of less than 0.05 was considered significant for two-tailed testing.

## 3 Results

### 3.1 Study population

**Figure 1:**
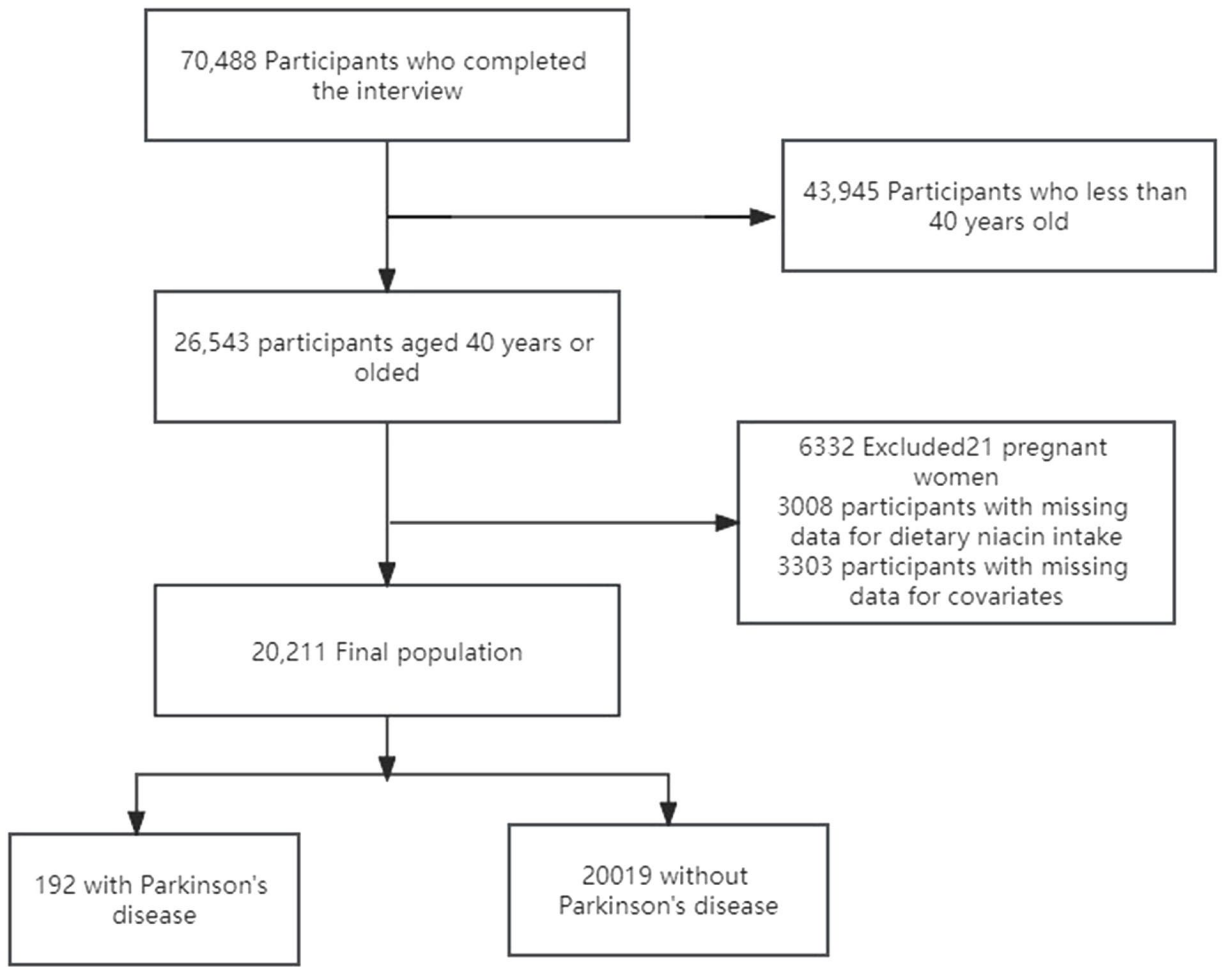
The study’s flow diagram.

This study screened data from 70,488 participants in seven cycles of NHANES surveys from 2005 to 2018. We excluded 43,945 individuals under the age of 40, 21 pregnant participants, 3,008 with missing niacin intake data, and 3,303 with missing covariate data. Ultimately, the study included 20,211 participants with complete data, among whom 192 had Parkinson’s disease. The exclusion and inclusion process is illustrated in Figure 1.

### 3.2 Demographic characteristics

Table 1 presents the baseline characteristics of all participants grouped according to the presence or absence of PD. A total of 192 patients (0.9%) had PD. The participants’ mean age ± SD was 59.5 ± 12.3 years, 10,308 (51%) were women, most of them were non-Hispanic white (9,432, 46.7%), and 50.8% had more than 12 years of education. The minimum daily niacin intake over a 24 h period was 0.002 mg, the maximum intake was 179.1 mg, and the average intake was 23.6 ± 11.7 mg. Individuals with PD may have exhibited the following characteristics: older age, non-Hispanic white ethnicity, lower household income, greater use of dietary supplements, combined hypertension, lower protein consumption, and lower niacin intake. Sex, education, marital status, BMI, smoking status, diabetes, coronary heart disease, total calories, carbohydrates, and fat consumption did not differ in the classification of PD.

**Table 1:** Population characteristics by categories of PD.

Characteristic	Total (*n* = 20,211)	No PD (*n* = 20,019)	PD (*n* = 192)	*p*-value
Sex, *n* (%)				0.991
Male	9,903 (49.0)	9,809 (49)	94 (49)	
Female	10,308 (51.0)	10,210 (51)	98 (51)	
Age (year), Mean (SD)	59.5 ± 12.3	59.5 ± 12.2	66.1 ± 12.2	<0.001
Race/ethnicity, *n* (%)				<0.001
Non-Hispanic white	9,432 (46.7)	9,304 (46.5)	128 (66.7)	
Non-Hispanic black	4,356 (21.6)	4,328 (21.6)	28 (14.6)	
Mexican American	2,763 (13.7)	2,747 (13.7)	16 (8.3)	
Others	3,660 (18.1)	3,640 (18.2)	20 (10.4)	
Education level (year), *n* (%)				0.553
<9	2,430 (12.0)	2,405 (12)	25 (13)	
9–12	7,517 (37.2)	7,440 (37.2)	77 (40.1)	
>12	10,264 (50.8)	10,174 (50.8)	90 (46.9)	
Marital status, *n* (%)				0.409
Married or living with a partner	12,581 (62.2)	12,467 (62.3)	114 (59.4)	
Living alone	7,630 (37.8)	7,552 (37.7)	78 (40.6)	
Family income, *n* (%)				0.003
Low	5,832 (28.9)	5,759 (28.8)	73 (38)	
Medium	7,722 (38.2)	7,647 (38.2)	75 (39.1)	
High	6,657 (32.9)	6,613 (33)	44 (22.9)	
Dietary supplements taken, *n* (%)	11,584 (57.3)	11,456 (57.2)	128 (66.7)	0.008
Smoking status, *n* (%)				0.981
Never	10,341 (51.2)	10,242 (51.2)	99 (51.6)	
Current	6,129 (30.3)	6,072 (30.3)	57 (29.7)	
Former	3,741 (18.5)	3,705 (18.5)	36 (18.8)	
Body mass index (kg/m^2^), *n* (%)				0.65
<25	5,083 (25.1)	5,032 (25.1)	51 (26.6)	
≥25	15,128 (74.9)	14,987 (74.9)	141 (73.4)	
Hypertension, *n* (%)	8,040 (39.8)	7,932 (39.6)	108 (56.2)	<0.001
Diabetes, *n* (%)	3,747 (18.5)	3,704 (18.5)	43 (22.4)	0.167
Coronary heart disease, *n* (%)	1,222 (6.0)	1,204 (6)	18 (9.4)	0.052
Stroke, *n* (%)	1,108 (5.5)	1,087 (5.4)	21 (10.9)	<0.001
Calorie consumption (kcal/d), Mean ± SD	1955.0 ± 798.0	1955.4 ± 798.3	1917.7 ± 766.7	0.515
Protein consumption (g/d), Mean ± SD	77.1 ± 34.0	77.2 ± 34.0	71.9 ± 29.7	0.031
Carbohydrate consumption (g/d), Mean ± SD	236.3 ± 101.5	236.2 ± 101.5	239.7 ± 101.7	0.643
Fat consumption (g/d), Median (IQR)	68.4 (48.6, 94.0)	68.4 (48.6, 94.0)	67.8 (48.4, 89.7)	0.736
niacin consumption (mg/d), Mean ± SD	23.6 ± 11.7	23.6 ± 11.7	21.4 ± 8.7	0.009

Fat consumption expressed as median (IQR) and rest continuous variables are expressed as mean ± SD.

### 3.3 Relationship between dietary niacin intake and PD risk

**Figure 2:**
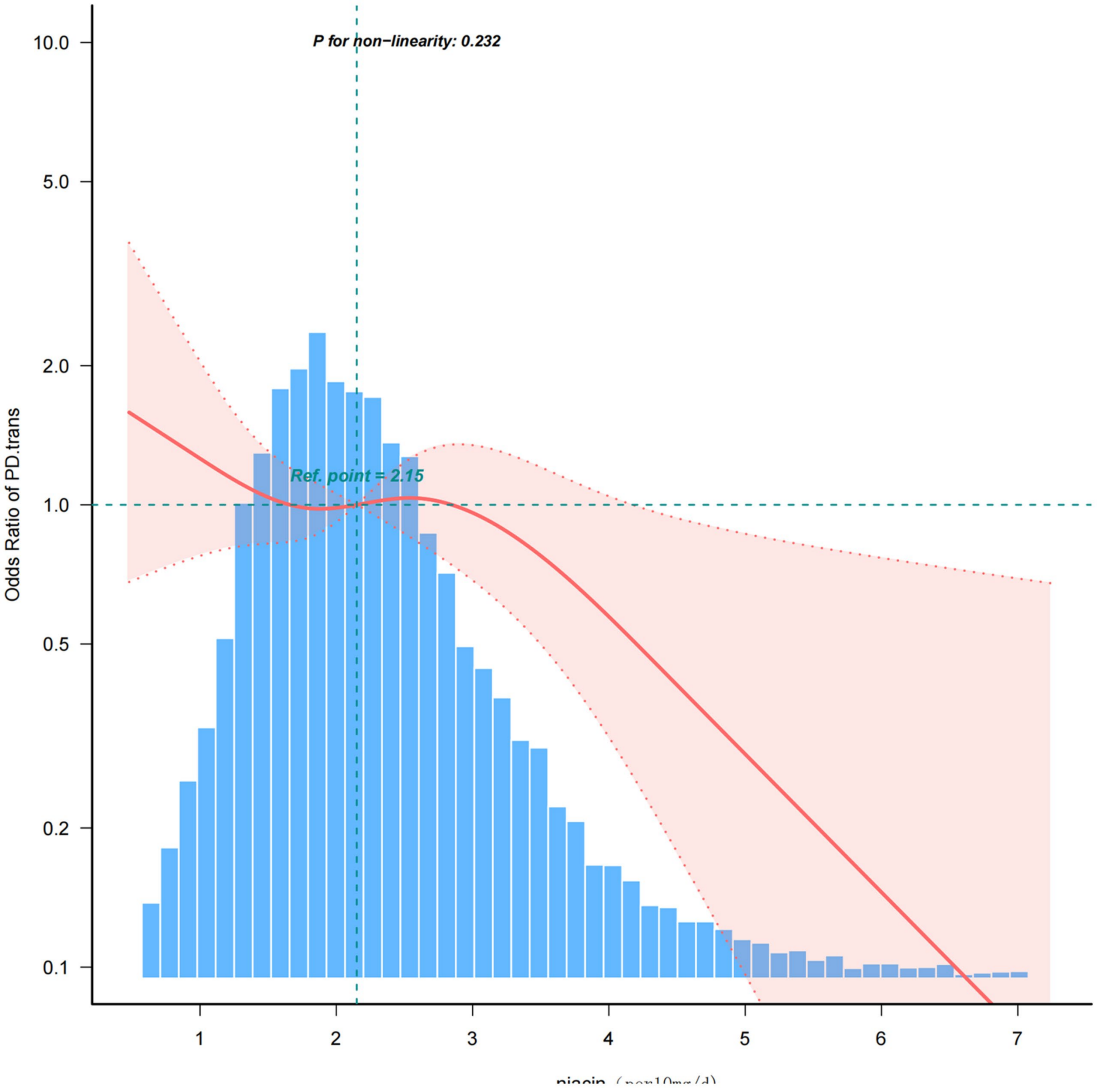
Association between dietary niacin intake and PD odds ratio. The solid and dashed lines represent the predicted value and the 95% confidence interval. They adjusted for age, sex, race, marital status, family income, education level, smoking status, body mass index, stroke, hypertension, coronary heart disease, diabetes, energy consumption, carbohydrate consumption, fat consumption, protein consumption, and whether they took dietary supplements. Only 99.5% of the data is displayed.

The univariate analysis demonstrated that age, race, family income, hypertension, protein consumption, and dietary supplements were associated with the risk of PD (Table 2). The results of multivariate logistic proportional risk regression analysis of the relationship between dietary niacin intake and the risk of PD are shown in Table 3. In models without adjustment for covariates, we found a significant independent inverse association between dietary niacin and the risk of PD (OR: 0.83, 95%CI: 0.72–0.95; *p* = 0.009). After adjusting for uncontrollable demographic characteristics variables (gender, age, and race) in Model 1, the inverse association between dietary niacin and the risk of developing PD did not change (OR: 0.84, 95%CI: 0.72–0.98; *p* = 0.027), and the difference was still statistically significant. In Model 2, the inverse association between dietary niacin and PD risk remained after adjustment for all demographic characteristics and smoking, BMI, energy consumption, protein consumption, carbohydrate consumption, and fat consumption (OR: 0.77, 95%CI: 0.6–0.99; *p* = 0.04). On the basis of Model 2, chronic comorbidities (diabetes, hypertension, coronary heart disease, and stroke) were added to Model 3 as a comprehensive adjustment of covariates, and the adjusted results showed that the inverse association between dietary niacin and PD risk remained stable. For every 10 mg/day increase in dietary niacin intake, the risk of PD was reduced by 23% (OR: 0.77, 95%CI: 0.6–0.99; *p* = 0.042). The RCS for the association between dietary niacin intake and the risk of PD is shown in Figure 2. Dietary niacin intake was inversely associated with PD risk when all confounding covariates were considered (nonlinearity, *p* = 0.232).

**Table 2:** Association of covariates and PD risk.

Variables	OR (95%CI)	*p*-value	Variables	OR (95%CI)	*p*-value
Sex, *n* (%)			Body mass index (kg/m^2^), *n* (%)		
Male	1 (reference)		<25	1 (reference)	
Female	1 (0.75–1.33)	0.991	≥25	0.93 (0.67–1.28)	0.65
Age (years)			Hypertension, *n* (%)		
40–65	1 (reference)		No	1 (reference)	
>65	2.55 (1.91–3.4)	<0.001	Yes	1.96 (1.47–2.61)	<0.001
Race/ethnicity, *n* (%)			Diabetes, *n* (%)		
Non-Hispanic white	1 (reference)		No	1 (reference)	
Non-Hispanic black	0.47 (0.31–0.71)	<0.001	Yes	1.27 (0.9–1.79)	0.168
Mexican American	0.42 (0.25–0.71)	0.001	Coronary heart disease, *n* (%)		
Others	0.4 (0.25 ~ 0.64)	<0.001	No	1 (reference)	
Education level (year), *n* (%)			Yes	1.62 (0.99–2.64)	0.054
<9	1 (reference)		Stroke, *n* (%)		
9–12	1 (0.63–1.57)	0.985	No	1 (reference)	
>12	0.85 (0.55–1.33)	0.478	Yes	2.14 (1.35–3.38)	0.001
Marital status, *n* (%)			Calorie consumption (kcal/d), Median (IQR)	1 (1–1)	0.515
Married or living with a partner	1 (reference)		Protein consumption (g/d), Median (IQR)	0.99 (0.99–1)	0.03
Living alone	1.13 (0.85–1.51)	0.41	Carbohydrate consumption (g/d), Median (IQR)	1 (1–1)	0.643
Family income, *n* (%)			Fat consumption (g/d), Median (IQR)	1 (1–1)	0.802
Low	1 (reference)		Niacin consumption (per 10 mg/d), Median (IQR)	0.83 (0.72–0.95)	0.009
Medium	0.77 (0.56–1.07)	0.121	Dietary supplements taken, *n* (%)	1.49 (1.11–2.02)	0.009
High	0.52 (0.36–0.76)	0.001			
Smoking status, *n* (%)					
Never	1 (reference)				
Current	0.97 (0.7–1.35)	0.861			
Former	1.01 (0.69–1.47)	0.979			

**Table 3:** The logistic regression of dietary niacin intake associated with PD risk.

Variable	*n*.total	*n*.event_%	Niacin (per 10 mg/d)	
			OR (95%CI)	*p*-value
Unadjusted	20,211	192 (0.9)	0.83 (0.72–0.95)	0.009
Model 1	20,211	192 (0.9)	0.84 (0.72–0.98)	0.027
Model 2	20,211	192 (0.9)	0.77 (0.6–0.99)	0.040
Model 3	20,211	192 (0.9)	0.77 (0.6–0.99)	0.042

Model 1: adjusted for RIAGENDR, age, and race.

Model 2: adjusted for RIAGENDR, age, race, edu, marital, PIR, supp, smoke, energy, protein, carbohydrate, fat, and BMI.

Model 3: adjusted for RIAGENDR, age, race, edu, marital, PIR, supp, smoke, energy, protein, carbohydrate, fat, BMI, hypertension, DM, CHD, and stroke.

CI, confidence interval; OR, odds ratios.

### 3.4 Subgroup analyses

**Figure 3:**
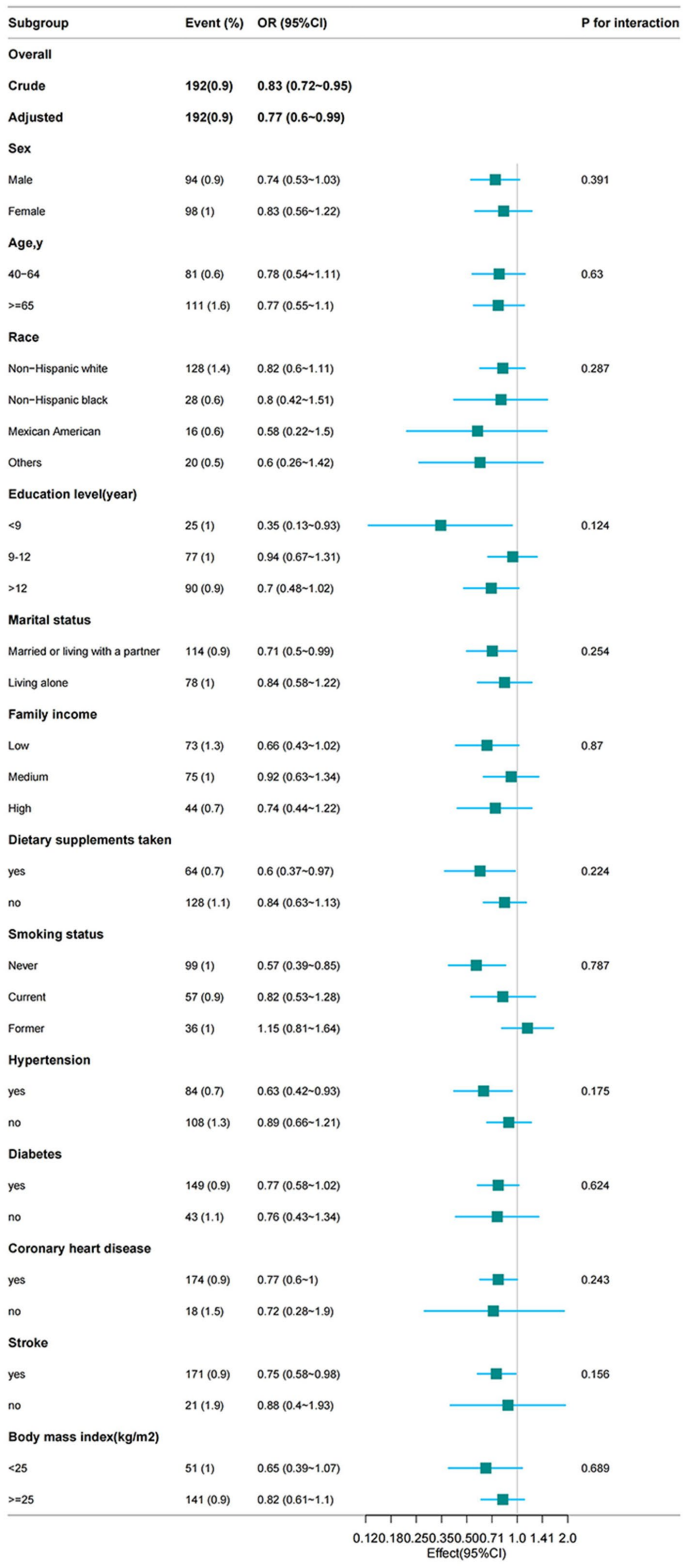
From the basic characteristics, the relationship between dietary niacin intake and PD is not only stratified components itself, each stratification factor was adjusted for all other variables (age, sex, marital status, ethnic group, education level, household income, smoking status, hypertension, diabetes, stroke, coronary heart disease, body mass index, energy expenditure, protein expenditure, carbohydrate expenditure, fat expenditure, dietary supplement use).

To determine whether the association between dietary niacin intake and the risk of PD was consistent across subgroups, we performed stratification and interaction analyses. When stratified by sex, age, race, marital status, education, smoking status, family income, BMI, and dietary supplements, hypertension, coronary heart disease, stroke, and diabetes, as shown in Figure 3, dietary niacin intake was more strongly associated with the risk of PD in those with fewer years of schooling (OR: 0.35, 95%CI: 0.13–0.93), married or cohabiting (OR: 0.71, 95%CI: 0.5–0.99), those taking dietary supplements (OR: 0.6, 95%CI: 0.37–0.97), non-smokers (OR: 0.57, 95%CI: 0.39–0.85), those with hypertension (OR: 0.63, 95%CI: 0.63–0.95), coronary heart disease (OR: 0.77, 95%CI: 0.6–1), and stroke (OR: 0.75, 95%CI: 0.88–0.98). Therefore, dietary niacin intake was an important protective factor for people with fewer years of education, married or cohabitating, taking dietary supplements, non-smokers, and those with hypertension, coronary heart disease, and stroke. When testing for interactions between subgroups using likelihood ratio tests, we found no statistically significant interactions in any subgroup.

### 3.5 Sensitivity analyses

After excluding the individuals with extreme energy consumption, 20,022 individuals left, and the association between dietary niacin intake and the risk of PD remained stable. In the fully adjusted model, the OR value for dietary niacin intake and PD risk was 0.77 (95%CI, 0.6–0.99, *p* = 0.042) (Table 4).

**Table 4:** Association between dietary niacin intake and PD risk in participants with extreme energy consumption was not included.

Variable	*n*.total	*n*.event_%	Niacin (per 10 mg/d)	
			OR (95%CI)	*p*-value
Unadjusted	20,022	190 (0.9)	0.82 (0.71–0.95)	0.009
Model 1	20,022	190 (0.9)	0.83 (0.71–0.98)	0.026
Model 2	20,022	190 (0.9)	0.77 (0.6–0.99)	0.041
Model 3	20,022	190 (0.9)	0.77 (0.6–0.99)	0.042

Model 1: adjusted for RIAGENDR, age, and race.

Model 2: adjusted for RIAGENDR, age, race, edu, marital, PIR, supp, smoke, energy, protein, carbohydrate, fat, and BMI.

Model 3: adjusted for RIAGENDR, age, race, edu, marital, PIR, supp, smoke, energy, protein, carbohydrate, fat, BMI, hypertension, DM, CHD, and stroke.

CI, confidence interval; OR, odds ratios.

## 4 Discussion

Based on NHANES data from 2005 to 2018, we investigated the relationship between dietary niacin intake and Parkinson’s disease (PD) among adults aged 40 and above in the United States. We found that PD patients had lower dietary niacin intake, and there was an association between niacin intake levels and PD risk. Both univariate and multivariate logistic regression analyses showed a negative correlation between dietary niacin intake and PD risk. Subgroup analyses revealed that dietary niacin intake was an important protective factor for individuals with shorter educational duration, married or cohabitating status, dietary supplement use, non-smoking status, as well as those with hypertension, coronary heart disease, and stroke. Additionally, restricted cubic spline (RCS) analysis indicated no nonlinear association between dietary niacin intake and PD.

Ender et al. (30) found that patients with PD may have chronic vitamin B3 deficiencies. Vascellari et al. (31) also found a decrease in B vitamins (B3 and B5) when studying the gut microbiota of patients with PD. Motawi et al. (32) evaluated the therapeutic effect of niacin on mouse models of PD through behavioral, biochemical, genetic, and histopathological observations, and found that food supplements containing niacin were effective in the treatment of PD. A randomized, double-blind trial in the United Kingdom showed that niacin supplementation might maintain or improve quality of life in people with PD and slow progression of the disease (16). Similarly, a randomized, double-blind controlled trial of U.S. military veterans showed that supplementation with low-dose niacin as adjunct therapy in patients with PD reduced neuroinflammation and improved motor function (15). The aforementioned studies all suggest the therapeutic significance of niacin for patients with PD. However, it is worth noting that there is currently a lack of large-scale clinical studies investigating the relationship between dietary niacin and Parkinson’s disease (PD) in the general population. To our knowledge, this study is the first to evaluate the association between dietary niacin intake and the risk of developing PD in US adults. This study included a general population sample from the United States, which was nationally representative. Our results suggest that higher dietary niacin intake may be associated with a reduced risk of PD in the US population, consistent with previous research findings. Earlier research also found that PD patients experienced controlled motor symptoms after taking high doses of niacin (500–2,000 mg/day), but they also encountered nightmares and rashes (33). In our study, the maximum daily dietary intake of niacin was 179.1 mg/day, so no safety issues related to excessive niacin intake were observed.

What is special about this study is the inclusion of total energy consumption, fat consumption, carbohydrate consumption, and protein consumption as covariates. Qu et al. (34) conducted a systematic review using the Embase and PubMed databases, concluding that high total energy consumption is associated with an increased risk of PD, and dietary fat consumption influences the risk of PD. Palavra et al. (35) found that PD patients reported higher total carbohydrate consumption. Kacprzyk et al. (36) searched four databases (Cochrane, PubMed, Embase, and Web of Science) and included 49 studies in their systematic review, analyzing the prevalence of malnutrition in PD patients, concluding that the prevalence or risk of malnutrition in the PD group is significant. Based on these studies, considering that total energy consumption, fat consumption, carbohydrate consumption, and protein consumption may all be related to the risk of PD, these factors were included as covariates in the study. The results showed that after comprehensive adjustment for total energy consumption, fat consumption, carbohydrate consumption, and protein consumption, the inverse relationship between dietary niacin intake and PD risk remained stable.

Based on previous studies, it is suggested that niacin is involved in the pathophysiological processes of PD via multiple mechanisms. First, chronic oxidative stress leads to oxidative damage to neuronal cell lipids, proteins, and DNA, resulting in the degeneration of substantia nigra dopaminergic neurons (37, 38). Degeneration and loss of dopaminergic neurons are the primary factors that contribute to PD progression (5, 39, 40). Experimental studies in various PD models have shown that niacin can improve oxidative stress associated with PD. Zhou et al. found that intraperitoneal injections of NADPH in an 1-methyl-4-phenyl-5-tetrahydropyridine (MPTP) animal model elevated glutathione levels and reduced the production of reactive oxygen species (ROS) (41). Qin et al. confirmed that exogenous NADPH possesses antioxidant activity both *in vivo* and in primary neuronal cultures (42, 43). In an animal model, Motawi et al. discovered that niacin decreased malondialdehyde and increased glutathione levels, thus reducing oxidative stress (32). Ganji et al. confirmed that niacin could increase NADP levels, inhibit the generation of ROS, and reduce glutathione levels, thereby reducing oxidative stress in endothelial cells (44).

Second, mitochondrial dysfunction has also been implicated in the pathogenesis of PD. Disruptions in mitochondrial dynamics (fission, fusion, transport, autophagy, etc.), complex I inhibition of the electron transport chain (ETC), and bioenergetic defects have all been confirmed to be associated with the pathogenesis of PD (45). The absence of niacin, an important cofactor in mitochondrial oxidative phosphorylation, is directly associated with mitochondrial dysfunction (12, 13).

In addition, a large number of studies have confirmed the link between neuroinflammation and PD. In patients with PD, inflammatory mediators such as TNF, IL-1β, IL-6, and IFNγ have been found in the cerebrospinal fluid and pathological findings of the dense part of the substantia nigra (46, 47). The niacin anti-inflammatory mechanism is mediated through the receptor GPR109A. Macrophages polarize from the M1 (pro-inflammatory) to the M2 (anti-inflammatory) phenotype through GPR109A (48). Neuroinflammation can be reduced by targeting GPR109A, thereby reducing the incidence of PD (7). Evidence shows that exogenous NADPH inhibits oxidative stress and glial cell-mediated neuroinflammation (41). In the MPTP model, the niacin metabolite NADPH effectively reduced MPP+-induced reactive oxygen species (ROS), p38 phosphorylation, and excessive production of cyclooxygenase-2 (COX2) inflammatory proteins, and inhibited glia-mediated neuroinflammation (41). Wakade et al. showed that in patients with PD, supplementation with low doses of niacin promoted anti-inflammatory processes and inhibited inflammation (48).

In summary, niacin may alter the pathology of PD through various neuroprotective mechanisms, including the reduction of oxidative stress, improvement of mitochondrial function, and amelioration of neuroinflammation.

This study had some limitations. First, owing to the limitations of the cross-sectional survey, we cannot infer causality from the results (49); therefore, further longitudinal research is necessary. Second, the NHANES uses anti-Parkinson drugs to define PD patients with PD, and cannot exclude sample inclusion for confounding reasons. In addition, in this study, we found that the confidence intervals for our conclusions were wide (0.6–0.99), suggesting that our sample size might be insufficient or the data variability might be high, thus necessitating cautious interpretation of these results. Future research requires larger sample sizes to obtain more precise estimates. Despite these uncertainties, our findings may still have practical significance in certain contexts, requiring careful consideration and balance in specific applications. Future research should aim to increase sample size and improve data quality to reduce the width of the confidence intervals, thereby providing more reliable evidence. Finally, there may be other confounding factors, such as physical activity, in the relationship between dietary niacin and PD. These additional factors should be considered in future studies to corroborate the findings of this research.

Since the NHANES dataset is nationally representative, our results can be generalized to the entire adult population of the United States to some extent. However, there may be differences for populations in other countries or regions due to variations in dietary habits, lifestyle, and genetic factors. Therefore, we recommend conducting similar studies in other regions to verify the external validity of these findings. Considering the limitations of this study, further research with larger sample sizes is needed to validate our results.

## 5 Conclusion

An inverse association between dietary niacin intake and the risk for PD was found in a large cross-sectional study of US adults aged 40 and older. For every 10 mg increase in dietary niacin intake, the risk of PD was reduced by 23%.

## Author contributions

LZ: Writing – original draft, Writing – review & editing, Data curation, Investigation, Methodology, Project administration, Resources, Software. SY: Formal analysis, Supervision, Validation, Writing – review & editing. XL: Data curation, Validation, Visualization, Writing – review & editing. CW: Data curation, Validation, Writing – review & editing. GT: Funding acquisition, Methodology, Writing – review & editing. XW: Formal analysis, Funding acquisition, Writing – review & editing. LL: Conceptualization, Methodology, Writing – review & editing.

## Footnotes

^1^https://www.cdc.gov/nchs/nhanes/index.htm accessed on 20 November 2023.
